# Region and dynamic specificities of adult neural stem cells and oligodendrocyte precursors in myelin regeneration in the mouse brain

**DOI:** 10.1242/bio.012773

**Published:** 2015-07-03

**Authors:** Béatrice Brousse, Karine Magalon, Pascale Durbec, Myriam Cayre

**Affiliations:** 1 Aix-Marseille Université, IBDM-UMR7288, Marseille 13288, France; 2 CNRS, IBDM-UMR7288, Marseille 13288, France

**Keywords:** Adult neural stem cells, Subventricular zone, Oligodendrocyte precursors, Myelin regeneration

## Abstract

Myelin regeneration can occur in the brain following demyelination. Parenchymal oligodendrocyte progenitors (pOPC) are known to play a crucial role in this process. Neural stem cells (NSC) residing in the ventricular-subventricular zone (V-SVZ) also have the ability to generate oligodendrocytes but their contribution to endogenous myelin repair was so far considered to be negligible. Here, we addressed the relative contribution of pOPC and V-SVZ-derived neural progenitors (SVZdNP) to remyelination in cuprizone mouse models of acute or chronic corpus callosum (CC) demyelination. Using genetic tracing, we uncover an unexpected massive and precocious recruitment of SVZdNP in the anterior CC after acute demyelination. These cells very quickly adopt an oligodendrocytic fate and robustly generate myelinating cells as efficiently as pOPC do. In more posterior areas of the CC, SVZdNP recruitment is less important whereas pOPC contribute more, underlining a regionalization in the mobilization of these two cell populations. Strikingly, in a chronic model when demyelination insult is sustained in time, SVZdNP minimally contribute to myelin repair, a failure associated with a depletion of NSC and a drastic drop of progenitor cell proliferation in V-SVZ. In this context, pOPC remain reactive, and become the main contributors to myelin regeneration. Altogether our results highlight a region and context-dependent contribution of SVZdNP to myelin repair that can equal pOPC. They also raise the question of a possible exhaustion of V-SVZ proliferation potential in chronic pathologies.

## INTRODUCTION

Since the discovery of ongoing neurogenesis throughout life in most mammals including humans, the idea that the adult brain may be endowed with regenerative capacities has been largely explored. Although most studies have been oriented toward neuronal regeneration, myelin regeneration is the most obvious example of endogenous spontaneous brain repair. Indeed, post-mortem observations of multiple sclerosis (MS) patient brains long since suggested that demyelination lesions could undergo spontaneous remyelination (Prineas and Connell, 1979). More recently, a large scale and systematic analysis showed that the extent of remyelination is highly variable, and can reach up to 90% of remyelination of global lesion area, even in patients with progressive disease (Patrikios et al., 2006). Much remains to be understood about this spontaneous repair process in order to design new therapeutic strategies promoting more efficient repair.

Rodent models of demyelination largely contributed to decipher the cellular mechanisms underlying remyelination. Parenchymal oligodendrocyte precursor cells (pOPC) were soon identified as the cell population responsible for myelin repair (Franklin et al., 1997; Gensert and Goldman, 1997). This was confirmed by the development of genetic tracing approaches (Zawadzka et al., 2010). After demyelinating insult, pOPC located close to the lesion proliferate, migrate and undergo terminal differentiation to replace lost oligodendrocytes. Beside, a number of studies suggested that neural stem cells (NSC) located in the ventricular-subventricular zone (V-SVZ) (Lim and Alvarez-Buylla, 2014) could also take part to this regenerative process (Aguirre et al., 2007; Cayre et al., 2006; Menn et al., 2006; Nait-Oumesmar et al., 1999; Picard-Riera et al., 2002). Indeed, although NSC in the adult V-SVZ mainly give rise to olfactory interneurons in the adult brain, a distinct NSC lineage generate oligodendroglial cells (Ortega et al., 2013) that migrate toward the corpus callosum (CC) and cortex (Menn et al., 2006). After demyelinating insult or when Wnt signaling is activated, this SVZ oligodendrogenesis is strongly increased (Menn et al., 2006; Ortega et al., 2013). Beside, after demyelination insult even SVZ-derived neuroblasts can generate new oligodendrocytes (Jablonska et al., 2010).

The relative contribution of these two sources of cells (pOPC and SVZdNP) endowed with different properties is unknown and may vary according to lesion localization and characteristics (acute or repeated/chronic demyelination).

In this study, we examined the respective contribution of pOPC and SVZdNP to remyelination in a mouse model of cuprizone-induced demyelination. This model offers the advantage to trigger a widespread demyelination of the CC with regional differences along the antero-posterior axis (Steelman et al., 2012), and to mimic either acute or chronic demyelination depending on treatment duration (Mason et al., 2004).

We show that SVZdNP are massively recruited to the CC during acute demyelination and that the majority of these recruited cells adopt an oligodendrocytic fate, thus contributing to the remyelination process. This contribution of SVZdNP is predominant in the anterior part of the CC whereas in more posterior areas the role of pOPC prevails. By contrast during long-term demyelination, SVZdNP recruitment to demyelinated CC drastically drops whereas pOPC remain reactive and keep generating new oligodendrocytes even though complete remyelination is not attained in the chronic model.

## RESULTS

NestinCre^ERT2^-YFP and PDGFRaCre^ERT2^-YFP mice were first treated with tamoxifen, to trigger recombination, hence inducing permanent YFP expression in SVZdNP or pOPC-derived lineages, respectively. Among the many different NestinCre^ERT2^ mouse lines, we decided to use the one generated by (Lagace et al., 2007) that shows the lowest recombination efficacy but the highest specificity to label neural progenitors in the adult brain, with only minor ependymal cell labeling (Sun et al., 2014; D. Lagace and J. Jankowsky, personal communications). Our results concerning SVZdNP mobilization are thus largely underestimated due to low recombination.

Before starting, we first checked that the time course of demyelination and remyelination in our transgenic mice was consistent with what is described for acute and chronic cuprizone models in the literature (supplementary material Fig. S1; for review, see Skripuletz et al., 2011).

### SVZdNP are massively mobilized toward the CC during acute demyelination

**Fig. 1.**
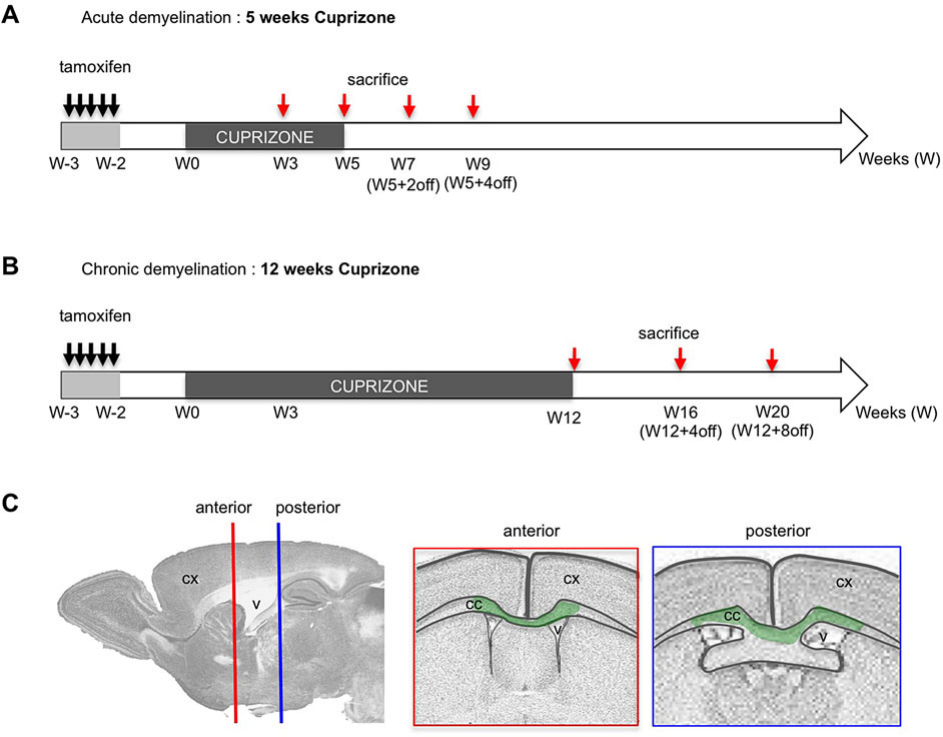
**Experimental design.** (A) Model of acute demyelination with short-term (5 weeks) cuprizone administration. Mice were injected for 5 consecutive days with tamoxifen to induce recombination 2 weeks before start of cuprizone treatment. Cuprizone was administered in food during 5 weeks. Mice were sacrificed at different time points during demyelination (W3 and W5) and during remyelination (W5+2off and W5+4off). (B) Model of chronic demyelination with long-term (12 weeks) cuprizone administration. Mice were injected for 5 consecutive days with tamoxifen to induce recombination 2 weeks before start of cuprizone treatment. Cuprizone was administered in food during 12 weeks. Mice were sacrificed at the end of cuprizone treatment (W12) and 4 and 8 weeks after the end of cuprizone treatment (W12+4off and W12+8off). (C) Areas analyzed. The corpus callosum (CC) was analyzed at two distinct locations: at an anterior level (in red, Bregma +0.5 to +1) and at a more posterior area at the level of the fornix (in blue, Bregma −0.3 to −0.8). cx, cortex; v, ventricle; cc, corpus callosum.

**Fig. 2.**
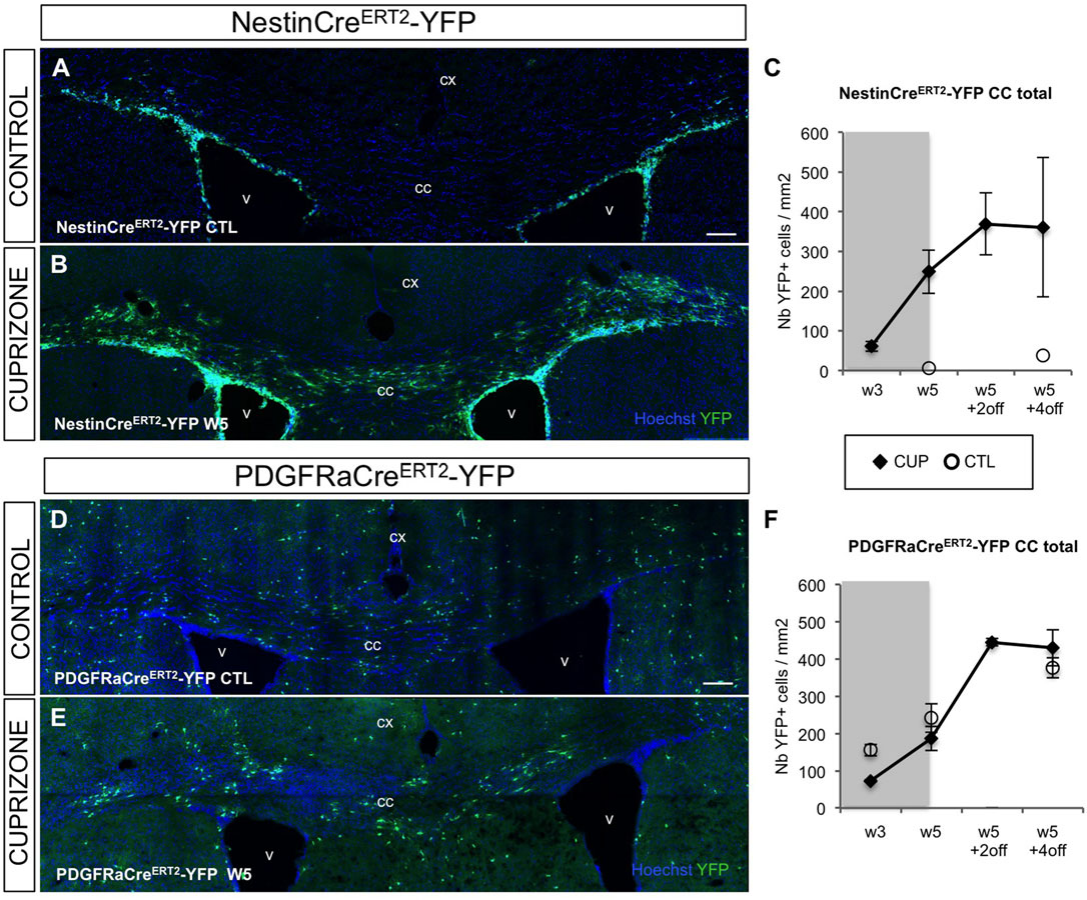
**Mobilization of SVZdNP and pOPC in the CC after short-term cuprizone-induced demyelination.** (A,B) YFP immunolabeling on coronal sections of the CC of (A) control (CTRL) or (B) cuprizone-treated (W5) NestinCre^ERT2^-YFP mice. While only rare YFP+ cells are observed in the CC of CTL mice, after 5 weeks cuprizone treatment the CC is colonized by numerous YFP+ cells. (C) Quantitative analysis of the YFP+ cell density in the CC at the different time points. (D,E) YFP immunolabeling on coronal sections of (D) the CC of control or (E) cuprizone-treated PDGFRaCre-YFP mice. Note the presence of YFP+ cells in the CC of CTL mice and the presence of cell clusters in mice at the end of the cuprizone treatment. (F) Quantitative analysis YFP+ cell density in the CC at the different time points. (C,F) CUP, cuprizone-treated; CTL, control. Gray block on the graph indicates the period of cuprizone administration. cx, cortex; v, ventricle; cc, corpus callosum. Scale bars 100 µm. Error bars=s.e.m.

Two weeks after tamoxifen injection, a cohort of both NestinCre^ERT2^-YFP and PDGFRaCre^ERT2^-YFP mice were fed with cuprizone for 5 weeks and analyzed during demyelination (W3), at the end of treatment (W5), 2 and 4 weeks after cuprizone removal (W5+2off; W5+4off) (Fig. 1A). In NestinCre^ERT2^-YFP control mice (injected with tamoxifen but not exposed to cuprizone), the density of YFP+ cells in the CC was very low (4.2±1.3 YFP+ cells/mm^2^ at W5 and 23.7±12.0 YFP+ cells/mm^2^ at W5+4off). By contrast, an unexpected massive recruitment of SVZdNP was observed in the CC of cuprizone-treated mice (Fig. 2A-C). Numerous YFP+ cells were present in the CC as early as W3 (Fig. 2C). YFP+ cell density exhibited a four-fold increase between W3 and W5 and reached a plateau 2 weeks after cuprizone removal (W5+2off) with 369.3±78.3 YFP+ cells/mm^2^ in the CC (Fig. 2C). This striking contrast in YFP+ cell density between control condition and demyelination highlights a very strong mobilization of V-SVZ cells from their niche toward the demyelinated CC.

In contrast to control NestinCre^ERT2^-YFP mice, numerous YFP+ cells were observed in the CC of control PDGFRaCre^ERT2^-YFP mice (Fig. 2D,F). This reflects the fact that physiologically, pOPC are resident in the CC irrespective of cuprizone treatment. YFP+ cell density in control PDGFRaCre^ERT2^-YFP mice steadily increased with time, which is consistent with basal level of pOPC proliferation in physiological condition. Furthermore, during the interval between tamoxifen injection and observation, a fraction of labeled cells matured into oligodendrocytes (59.0±2.9% of YFP+CC1+ cells 7 weeks after recombination, not shown). In PDGFRaCre^ERT2^-YFP mice YFP+ cell density is thus the result of a balance between labeled pOPC proliferation and labeled oligodendrocytes cell death, which complicates the interpretation. Nevertheless, after 3 weeks of cuprizone administration, YFP+ cell density was significantly lower in cuprizone-treated compared to control mice, reflecting cell death in the oligodendrocytic lineage (73.0±3.1 versus 159.6±3.6 cells/mm^2^; *P*<0.05). Despite this initial lowering, YFP+ cell density more than doubled between W3 and W5 although mice were still under cuprizone treatment and caught up control levels at W5; it increased even more after cuprizone withdrawal (Fig. 2F). While YFP+ cells were homogeneously disseminated in the CC of control mice, clusters of YFP+ cells were observed in cuprizone-treated mice, suggesting clonal pOPC expansion (Fig. 2E). Indeed, cell proliferation analysis revealed a 5-fold increase in the proportion of proliferating pOPCs after 5 weeks cuprizone treatment compared to non-treated mice (11.1±0.7 versus 3.2±0.9% of Ki67+ cells among YFP+NG2+ cells; *P*<0.05).

Comparison of both mouse lines reveals a stronger reactivity of SVZdNP than pOPC during the early phases (i.e. during demyelination) since between W3 and W5 YFP+ cell density is multiplied by 4 in NestinCre^ERT2^-YFP mice and only by 2.5 in PDGFRaCre^ERT2^-YFP mice. In agreement with this observation, cell proliferation at W5 was significantly higher in SVZdNP (16.7±1.2% of Ki67+ cells among YFP+ cells) than in pOPC (11.1±0.7%) (*P*=0.01; Table 1). After cuprizone removal, SVZdNP proliferation considerably decreased (Table 1) contributing to the attenuation of the slope of YFP+ cell density (Fig. 2C). By contrast, pOPC proliferation is less reduced after cuprizone removal leading to constant increase in cell density (Fig. 2F).

**Table 1.**
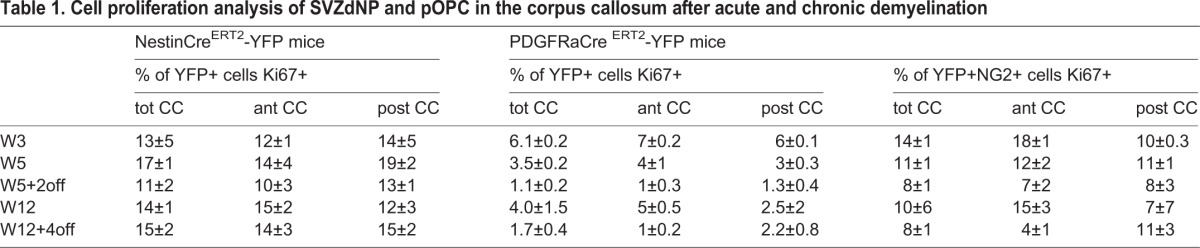
**Cell proliferation analysis of SVZdNP and pOPC in the corpus callosum after acute and chronic demyelination**

These results underline a surprising level of SVZdNP mobilization during acute cuprizone-induced demyelination. The dynamic of cell mobilization over time shows a strong mobilization of SVZdNP during the demyelination phase whereas pOPC mobilization is higher during the remyelination phase after cuprizone removal.

### SVZdNP and pOPC repopulate the demyelinated CC with a regionalized pattern

**Fig. 3.**
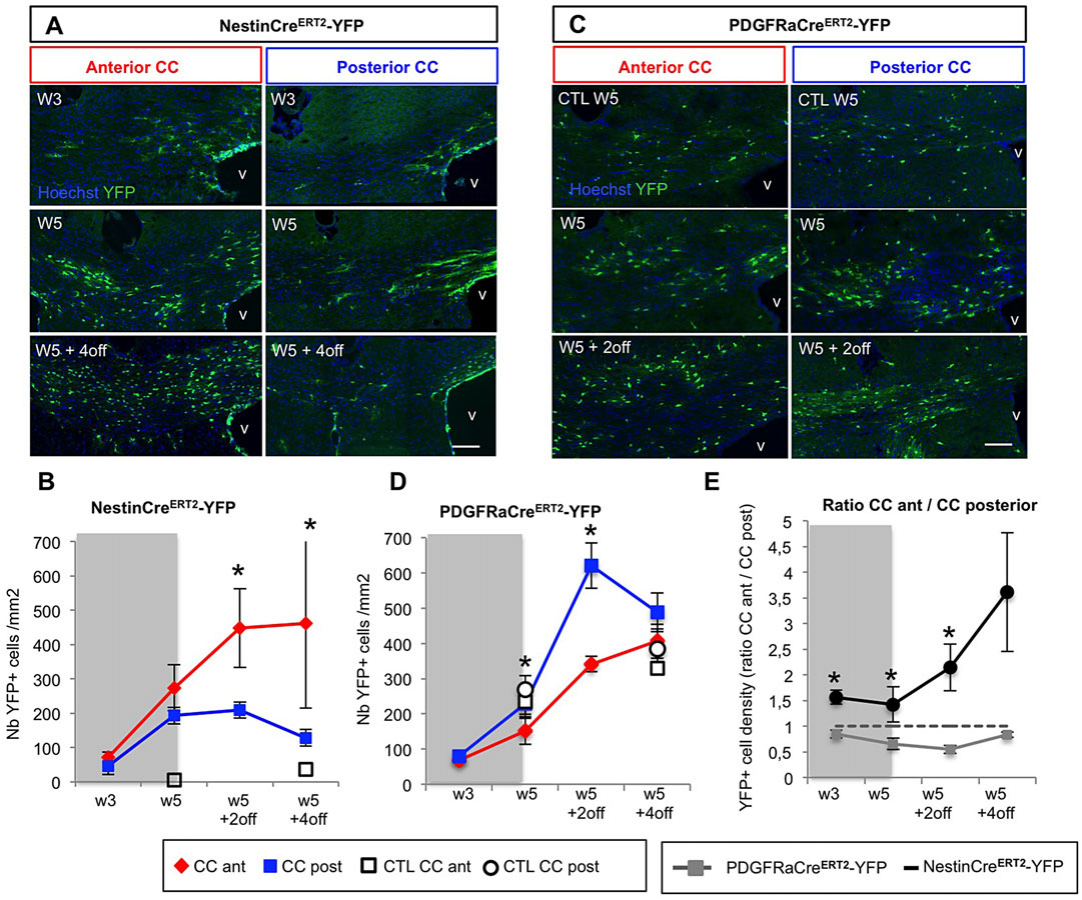
**Regionalized mobilization of SVZdNP and pOPC in the CC after short-term cuprizone-induced demyelination.** (A) YFP immunolabeling on coronal sections of NestinCre^ERT2^-YFP mice at different time points of cuprizone treatment, focused on the central part of the CC at anterior and posterior levels. Many YFP+ cells are present in the CC. Note the difference between anterior and posterior CC. (B) Quantitative analysis of YFP+ cell density in the CC of NestinCre^ERT2^-YFP mice. (C) YFP immunolabeling on coronal sections of PDGFRaCre^ERT2^-YFP mice at different time points of cuprizone treatment, focused on the central part of the CC at anterior and posterior levels. (D) Quantitative analysis of YFP+ cell density in the CC of PDGFRaCre^ERT2^-YFP mice. (E) Ratio of YFP+ cell density in anterior versus posterior CC in both mouse lines. In NestinCre^ERT2^-YFP mice this ratio is >1 whereas it is <1 in PDGFRaCre^ERT2^-YFP mice showing a regionalization in the contribution of SVZdNP and pOPC to CC repopulation. (B,D,E) CC ant (red), anterior CC; CC post (blue) posterior CC; CTL, control. Gray block on the graph indicates the period of cuprizone administration. Scale bars in A and C, 100 µm. Error bars=s.e.m.; *P<0.05.

Considering the widespread dissemination of pOPC compared to the localized V-SVZ niche, we then questioned if the contribution of the two sources of cells was homogeneous in the whole CC. We show that SVZdNP YFP+ cell density was significantly higher in the anterior part of the CC compared to more posterior areas after cuprizone removal (Figs 1C and 3A,B) (462.3±247.8 versus 128.1±35.1 YFP+ cells/mm^2^ in the anterior and posterior CC respectively at W5+4off; *P*=0.03). Conversely, pOPC contribution was significantly higher in posterior than in anterior CC (Fig. 3C,D; *P*<0.05), and thus by W5 YFP+ cell density had reached back control values in posterior CC but not yet in anterior CC (Fig. 3D). Finally, comparing the ratio of anterior versus posterior YFP+ cell density in each animal revealed a significant difference between the two mouse lines: while this ratio is superior to 1 in NestinCre^ERT2^-YFP mice, it is inferior to 1 in PDGFRaCre^ERT2^-YFP mice (Fig. 3E; *P*<0.05). These results reveal a regionalization in the mobilization of SVZdNP and pOPC during the repair process. In order to better understand mechanisms underlying such regionalization, we analyzed cell proliferation in anterior and posterior CC. No significant difference in SVZdNP proliferation was detected in anterior and posterior CC (Table 1), suggesting that higher cell density observed in anterior CC is the consequence of more abundant recruitment from the niche to anterior CC; once in the CC, SVZdNP show similar proliferation dynamic in both compartments. Unexpectedly, pOPC proliferation was slightly higher in anterior CC at early time point (W3), then this difference vanishes from W5 onward (Table 1). Therefore it does not seem that the regionalized repartition of pOPC in the acute model is linked to distinct proliferation properties.

### SVZdNP recruited to the demyelinated CC efficiently produce oligodendrocytes and contribute to myelin repair

**Fig. 4.**
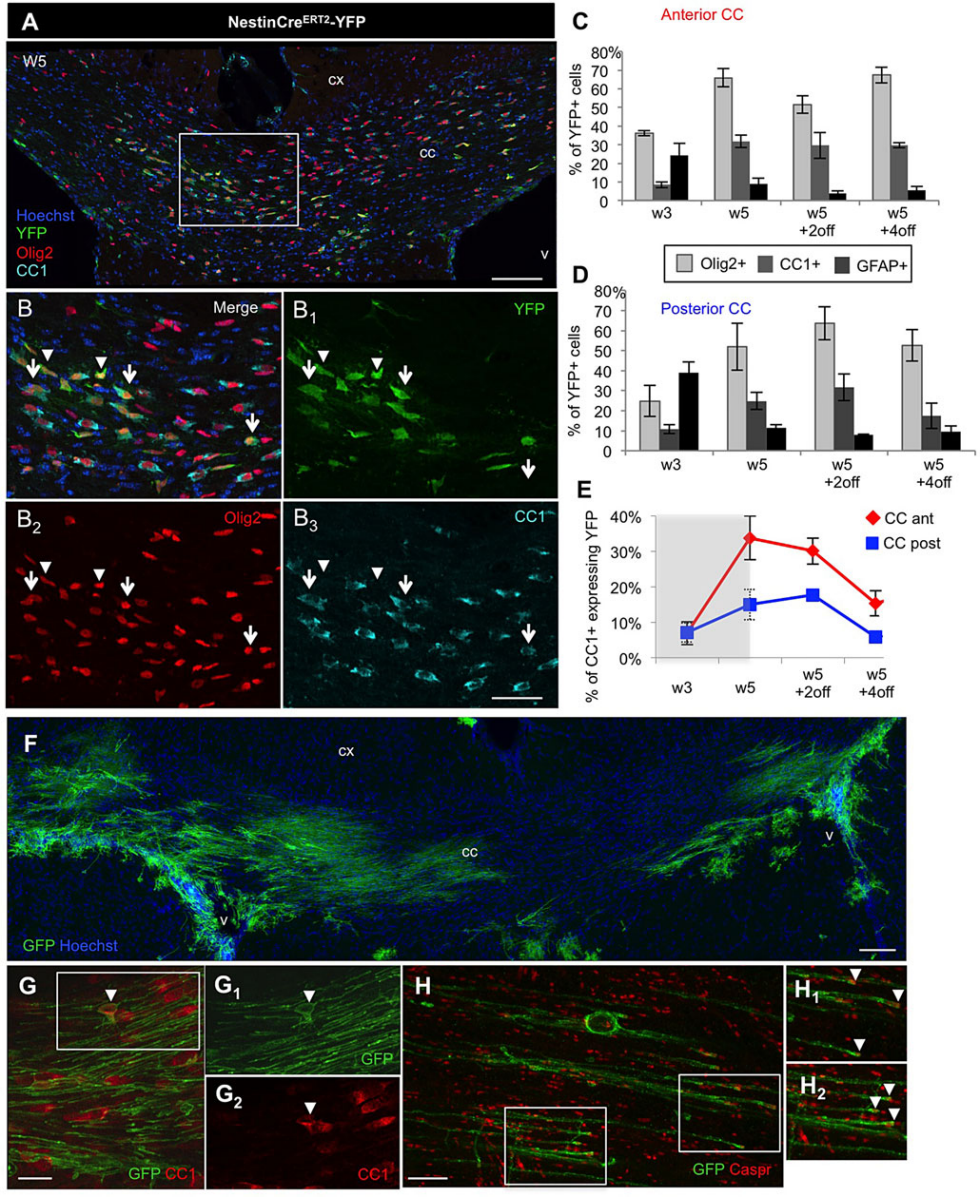
**Phenotypic analysis of YFP+ SVZ-derived cells in the CC during acute cuprizone-induced demyelination.** (A,B) Triple immunolabeling showing co-expression of YFP, Olig2 and CC1 in numerous cells after 5 weeks cuprizone administration. Arrowheads in B represent YFP+ cells expressing Olig2 but not CC1. Arrows represent YFP+ cells expressing both Olig2 and CC1. (C,D) Quantitative analysis of the proportion of YFP+ cells expressing Olig2, CC1 and GFAP in the (C) anterior CC and (D) posterior CC. (E) Proportion of total CC1+ cells expressing YFP in the CC. CC ant (red), anterior CC; CC post (blue) posterior CC. Gray block on the graph indicates the period of cuprizone administration. (F-H) Myelination potential of SVZdNP is visualized by using mTmG reporter mouse line. Note in F the presence of typical myelin segments. (G,H) immunolabeling showing co-expression of YFP and CC1 (arrowhead in G) and the paranodin labeling concentrate at the tip of GFP+ segments (arrows in H_1_ and H_2_). cx, cortex; v, ventricle; cc, corpus callosum. Scale bars: in A, 100 µm; in B, 50 µm; in F, 100 µm; in G, 20 µm and in H, 10 µm. Error bars=s.e.m.

Since SVZdNP are mostly destined to become neurons in physiological conditions, the phenotype of these YFP+ cells in the CC was examined by immunofluorescence (Fig. 4A,B). At early time points (W3), a significant fraction of YFP+ cells (up to almost 40% in the posterior CC) expressed the astrocytic marker GFAP (Fig. 4C,D and Table 2). This proportion decreased to around 10% later on. These observations suggest that SVZdNP take part to the important reactive astrogliogenesis that has been shown to occur concomitantly to oligodendrocyte loss in the cuprizone model (Skripuletz et al., 2011). Regarding oligodendrocyte lineage, at W3 more than one third (36.1±1.3%) of YFP+ SVZdNP already expressed the Olig2 marker in the anterior CC (Fig. 4A-C and Table 2) and this proportion reached 66.0±4.8% as soon as W5. The oligodendrocytic fate of SVZdNP YFP+ cells in the posterior and anterior CC did not differ significantly (see Fig. 4C,D and Table 2). These results indicate that SVZdNP promptly adopt an oligodendrocytic fate once in the CC. We then used CC1 labeling as a marker of mature oligodendrocytes (Fig. 4B_3_). At early time points (W3), we already observed 10% of YFP+ cells expressing CC1 and this proportion reached up to 30% at later time points during the remyelination phase (Fig. 4C,D and Table 2). The proportion of YFP+Olig2+ cells that matured into YFP+Olig2+CC1+ oligodendrocytes steadily increased from W3 to W5+2off (Table 2). Remarkably, this production of new oligodendrocytes significantly contributed to repopulate the CC. Indeed, at the end of demyelination (W5) and during remyelination (W5+2off), among all CC1+ cells present in the anterior CC more than 30% were YFP+ indicating that one third of mature oligodendrocytes in this area are derived from V-SVZ (Fig. 4E). Consistent with the lower recruitment of SVZdNP observed in the posterior CC, only one sixth of all CC1+ cells were YFP+ in the posterior CC (15.0±4.3% at W5 and 17.7±1.0% at W5+2off). Interestingly, these proportions significantly decreased at W5+4off, possibly reflecting the contribution of cells from different origin (most likely pOPC) to the total pool of oligodendrocytes (Fig. 4E).

**Table 2.**
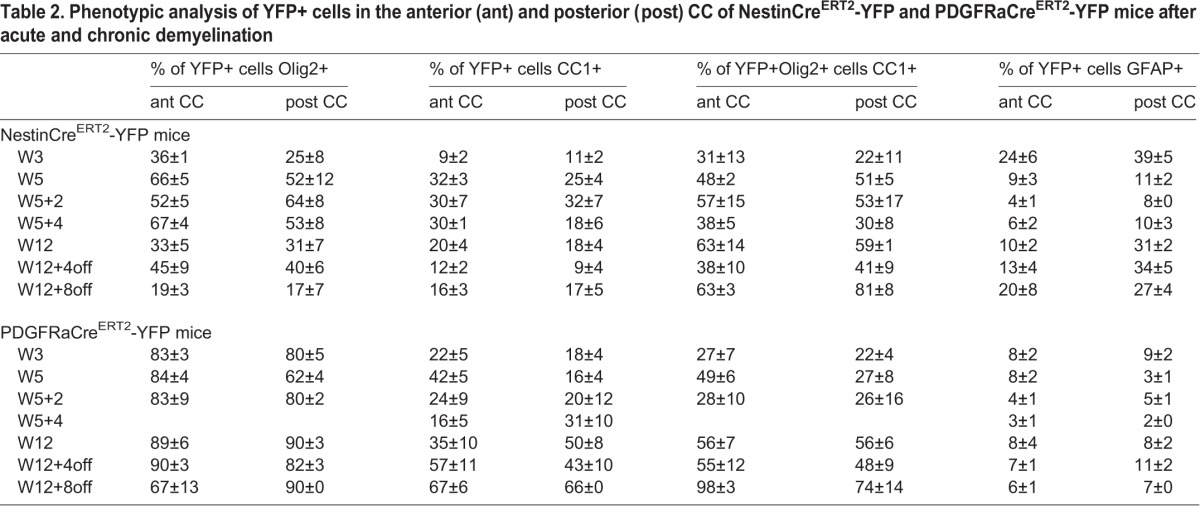
**Phenotypic analysis of YFP+ cells in the anterior (ant) and posterior (post) CC of NestinCre^ERT2^-YFP and PDGFRaCre^ERT2^-YFP mice after acute and chronic demyelination**

We next asked whether these V-SVZ-derived CC1+ cells efficiently undergo the terminal stages of differentiation (producing a myelin sheath) by using the mTmG reporter mouse line, another Rosa26-based reporter, which upon cre-mediated recombination expresses a myristylated GFP, thus enabling to visualize the myelin segments. V-SVZ-derived oligodendrocytes exhibited typical morphology of myelinating cells expressing CC1 (Fig. 4G) with long straight GFP+ segments (Fig. 4F) forming axoglial junctions at the nodes of Ranvier (Fig. 4H) supporting the hypothesis of new functional myelin generated by SVZdNP. Altogether these results demonstrate that SVZdNP largely contribute to myelin repair after acute cuprizone-induced demyelination.

**Fig. 5.**
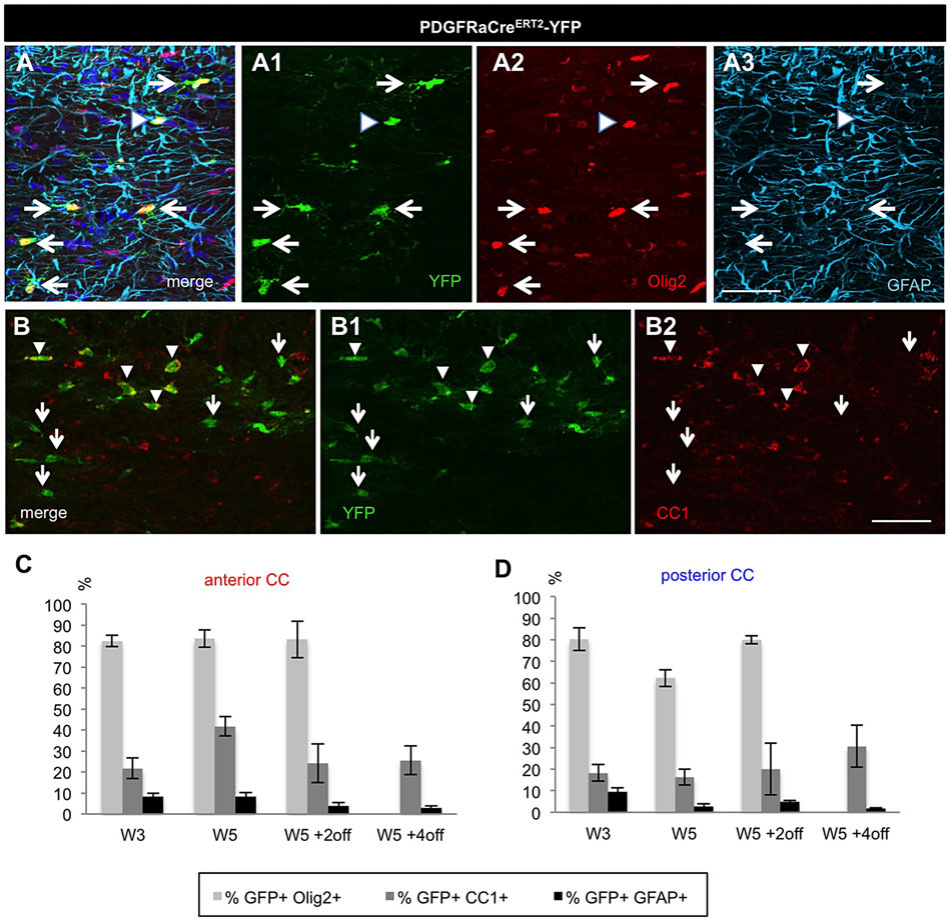
**Phenotypic analysis of pOPC-derived YFP+ cells in the CC during acute cuprizone-induced demyelination.** (A) Triple immunolabeling showing that almost all YFP+ cells are Olig2+ (arrows) and only very few cells also express the astrocytic marker GFAP (arrowhead). (B) Double immunolabeling illustrating CC1 expression among YFP+ cells in the CC (arrowhead), arrows show YFP+ cells that do not express CC1. (C,D) Quantitative analysis of the proportion of YFP+ cells expressing Olig2, CC1 and GFAP in the (C) anterior and (D) posterior CC. Scale bars, 50 µm. Error bars=s.e.m.

Several studies demonstrated that adult pOPC are restricted to the oligodendrocyte lineage in physiological conditions (Dimou et al., 2008; Kang et al., 2010; Rivers et al., 2008; Zhu et al., 2011). In our study, although the vast majority of YFP+ cells in the CC of PDGFRaCre^ERT2^-YFP mice expressed Olig2 and thus remained within the oligodendrocytic lineage (Fig. 5A,C,D; Table 2), up to 9% (at W3) of YFP+ cells expressed GFAP (Fig. 5A,C,D), a marker of astrocytes, suggesting some lineage plasticity in this pathological context. We then checked for oligodendrocyte maturation using CC1 labeling. Whereas in control mice the proportion of YFP+CC1+ cells among YFP+ cells was around 60% (59.0±2.9% in W5 controls, data not shown), it was reduced to around 20% after 3 weeks cuprizone treatment (Fig. 5B-D; Table 2). This proportion reached a maximum of 41.7±4.6% in the anterior CC (at W5) and always stayed below 30% in the posterior CC (Fig. 5B-D; Table 2), thus remaining below control levels. This lower proportion of differentiated cells is consistent with the increased pOPC proliferation induced by cuprizone. Furthermore newly formed oligodendrocytes may also be killed by ongoing treatment.

**Fig. 6.**
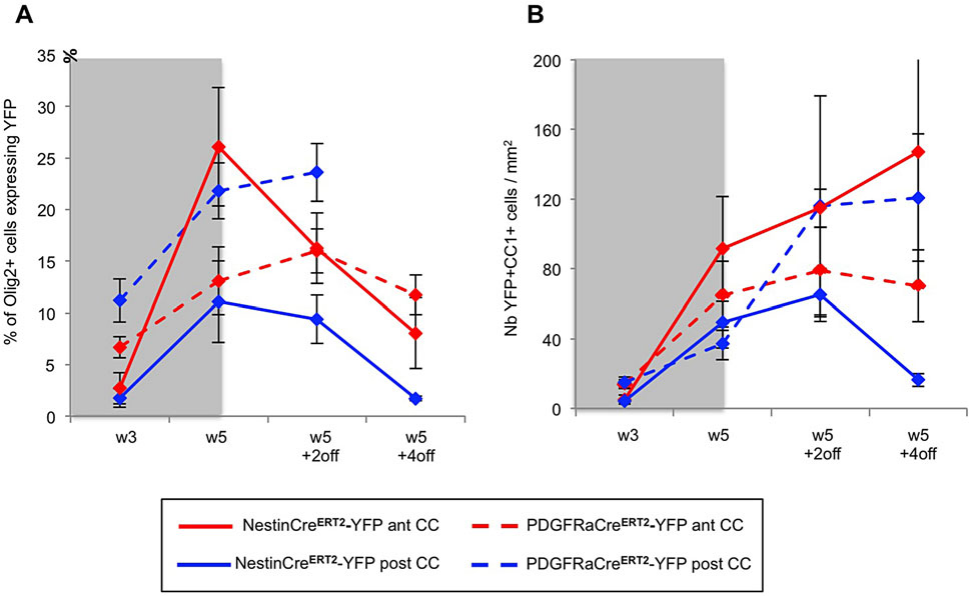
**Contribution of SVZdNP and pOPC to the oligodendrocytic cell population in the CC during acute cuprizone-induced demyelination.** (A) Proportion of Olig2+ cells expressing YFP in the anterior (red) and posterior (blue) CC of both mouse lines (NestinCre^ERT^2-YFP mice in plain lines and PDGFRaCre^ERT2^-YFP mice in dotted lines). (B) YFP+CC1+ cell density in the anterior (ant CC, red) and posterior (post CC, blue) CC of both mouse lines (NestinCre^ERT2^-YFP in plain lines and PDGFRaCre^ERT2^-YFP in dotted lines). Gray blocks on the graphs indicate the period of cuprizone administration. Error bars=s.e.m.

Altogether these results show that after short-term cuprizone administration both SVZdNP and pOPC are mobilized and contribute to the repair process. At the end of cuprizone administration (W5) their contribution to the total number of Olig2+ cells in the CC is quantitatively similar but with distinct antero-posterior distribution (Fig. 6A). The proportion of YFP+Olig2+ cells then decreases between W5 and W5+2off in the NestinCre^ERT^2-YFP mice whereas it keeps progressing in PDGFRaCre^ERT2^-YFP mice, suggesting an early and strong but transient mobilization of SVZdNP and a sustained pOPC contribution. Remarkably a lower proportion of YFP+Olig2+ cells mature into CC1+ oligodendrocytes in PDGFRaCre^ERT2^-YFP mice compared to NestinCre^ERT2^-YFP mice (Figs 4C,D and 5C,D), enlightening rapid and efficient oligodendrocytic differentiation capacities of SVZdNP. Overall, the final result is a comparable level of oligodendrogenesis from SVZdNP and pOPC in the acute model of demyelination but with different distribution along the CC (Fig. 6B).

### SVZdNP fail to contribute to the repair process after long-term exposure to cuprizone whereas pOPC are still mobilized

In the chronic model of cuprizone-induced demyelination, the regeneration process is known to be incomplete (Mason et al., 2004); indeed, we observed an inability to catch up control levels in myelin content and Olig2 cell density even 8 weeks after cuprizone removal (supplementary material Fig. S1). To evaluate the specific contribution of SVZdNP and pOPC in this context, mice were fed for 12 weeks with cuprizone and sacrificed at the end of treatment, 4 or 8 weeks after removal of cuprizone (Fig. 1B).

**Fig. 7.**
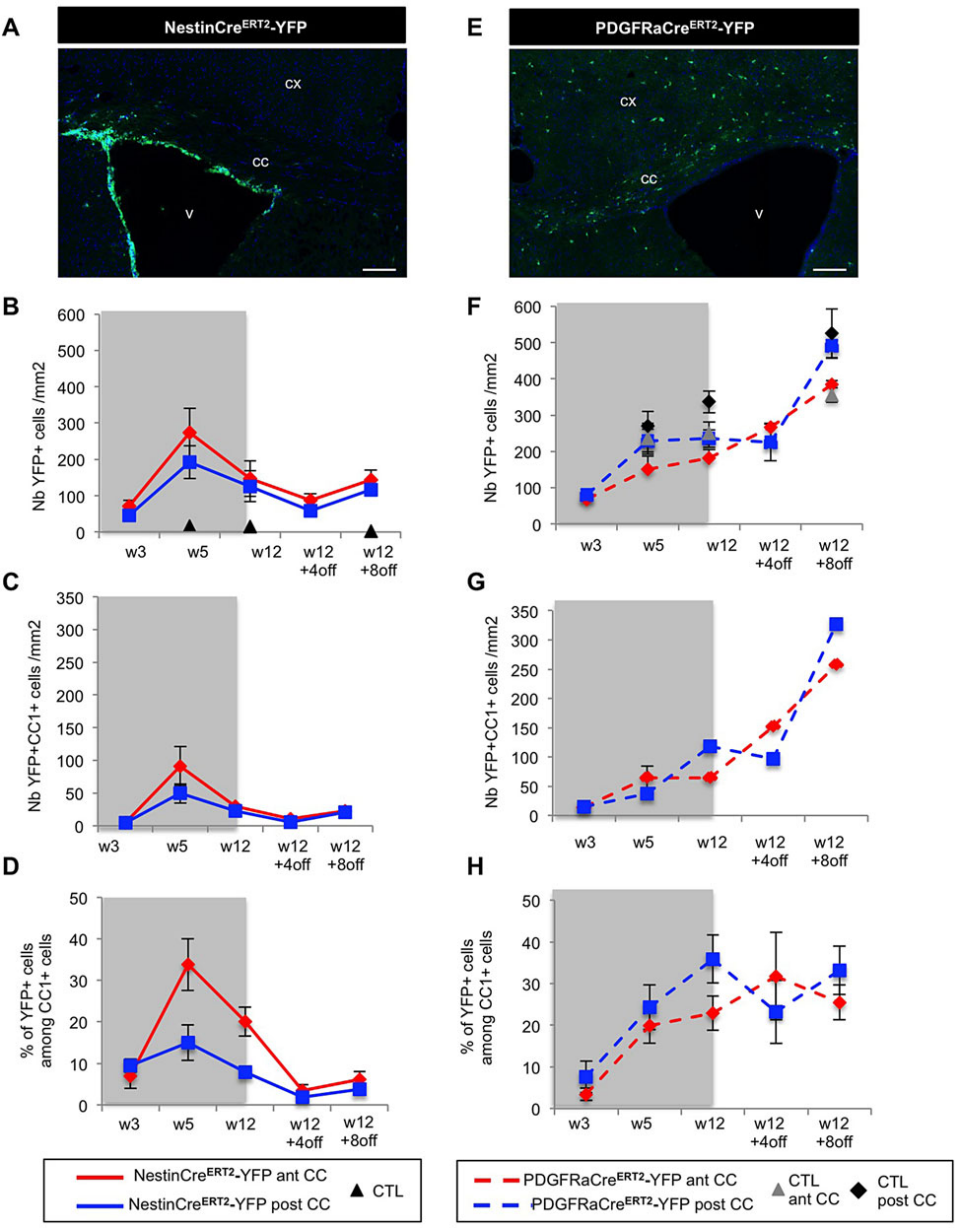
**Mobilization of SVZdNP and pOPC in the CC during long-term cuprizone-induced demyelination.** (A) YFP immunolabeling on coronal sections of the CC of NestinCre^ERT2^-YFP mice at W12+8. Note the presence of only rare cells in the CC. (B,C) Quantitative analysis of (B) YFP+ and (C) YFP+CC1+ cell density in anterior (ant CC, red) and posterior (post CC, blue) CC of NestinCre^ERT2^-YFP mice. (D) Contribution of SVZdNP to total CC1+ mature oligodendrocyte population in the anterior (red) and posterior (blue) CC. (E) YFP immunolabeling on coronal sections of the CC of PDGFRaCre^ERT2^-YFP mice at W12+8. (F,G) Quantitative analysis of (F) YFP+ and (G) YFP+CC1+ cell density in anterior (red) and posterior (blue) CC of PDGFRaCre^ERT2^-YFP mice. (H) Contribution of pOPC to total CC1+ mature oligodendrocyte population in the anterior (red) and posterior (blue) CC. Gray blocks on the graphs indicate the period of cuprizone administration. cx, cortex; v, ventricle; cc, corpus callosum. Scale bars in A and E, 100 µm. Error bars=s.e.m.

The analysis of NestinCre^ERT2^-YFP mice in this chronic demyelination paradigm (Fig. 7A-D) shows that following the strong initial mobilization between W3 and W5, sustained cuprizone administration resulted into an important decrease in the number of YFP+ cells in the anterior and posterior CC from W5 to W12 (Fig. 7B). Furthermore, YFP+ cell density also failed to increase after cuprizone removal even after 8 weeks of normal chow (Fig. 7B). Notably, regionalization between anterior and posterior CC was no longer obvious (Fig. 7B). Interestingly, although the total number of SVZdNP in the CC decreased severely, their capacity to proliferate was similar to that observed in the acute model (Table 1). Thus, we conclude that fewer numbers of progenitors were recruited from the niche to the CC after long-term cuprizone administration.

In addition to reduced mobilization of SVZdNP, their efficacy to produce differentiated oligodendrocytes was also significantly lower than in the acute context. We observed a lowering in the proportion of YFP+ cells expressing Olig2 and CC1 compared to the acute model. Indeed at W12+8off, only 18.6±2.6% of YFP+ cells in the anterior CC were also Olig2+ and 15.9±3.2% expressed CC1 (compared to 67.4±4.2% YFP+Olig2+ and 29.7±1.3% YFP+CC1+ at W5+4off; *P*=0.01 and 0.02 respectively, see also Table 2). Consequently, the total number of oligodendrocytes generated in the CC by SVZdNP after long-term cuprizone administration was very low (Fig. 7C). By contrast, the number of YFP+ cells in the CC of PDGFRaCre^ERT2^-YFP mice was stable during cuprizone administration from W5 to W12 and significantly increased after treatment termination (from 182.0±23.6 at W12 to 383.6±11.0 at W12+8; *P*=0.03; Fig. 7E,F). Notably, pOPC proliferation was not significantly altered after 12 weeks cuprizone administration compared to 5 weeks treatment (Table 1). Furthermore, unlike what we observed in NestinCre^ERT2^-YFP mice, the proportion of YFP+ cells maturing into CC1+ oligodendrocytes was significantly higher in the chronic compared to the acute model (Fig. 5C,D and Table 2). Indeed, 4 weeks after cuprizone removal (W12+4off) around half of YFP+ cells expressed CC1 and this proportion reached 67% at W12+8off (Table 2).

Thus in the cuprizone-induced chronic demyelination model, contrary to SVZdNP, pOPC keep contributing to the CC repopulation with new oligodendrocytes (Fig. 7G). The quantification of the respective contribution of SVZdNP and pOPC to total oligodendrocyte population in the CC after chronic demyelination shows that SVZdNP are well represented during the early step of demyelination but their contribution drastically drops with time when cuprizone is maintained for a long period (Fig. 7D). By contrast, the proportion of pOPC-derived oligodendrocytes keeps increasing from W3 to W12 and then reaches a plateau around 30% of total oligodendrocytes (Fig. 7H). In conclusion, under sustained demyelination insult, SVZdNP lose their ability to be mobilized toward demyelinated CC and to generate new oligodendrocytes. In such conditions, pOPC become the major source of mature oligodendrocytes, even if insufficient to achieve complete remyelination.

### Long-term cuprizone administration inhibits SVZ cell proliferation

**Fig. 8.**
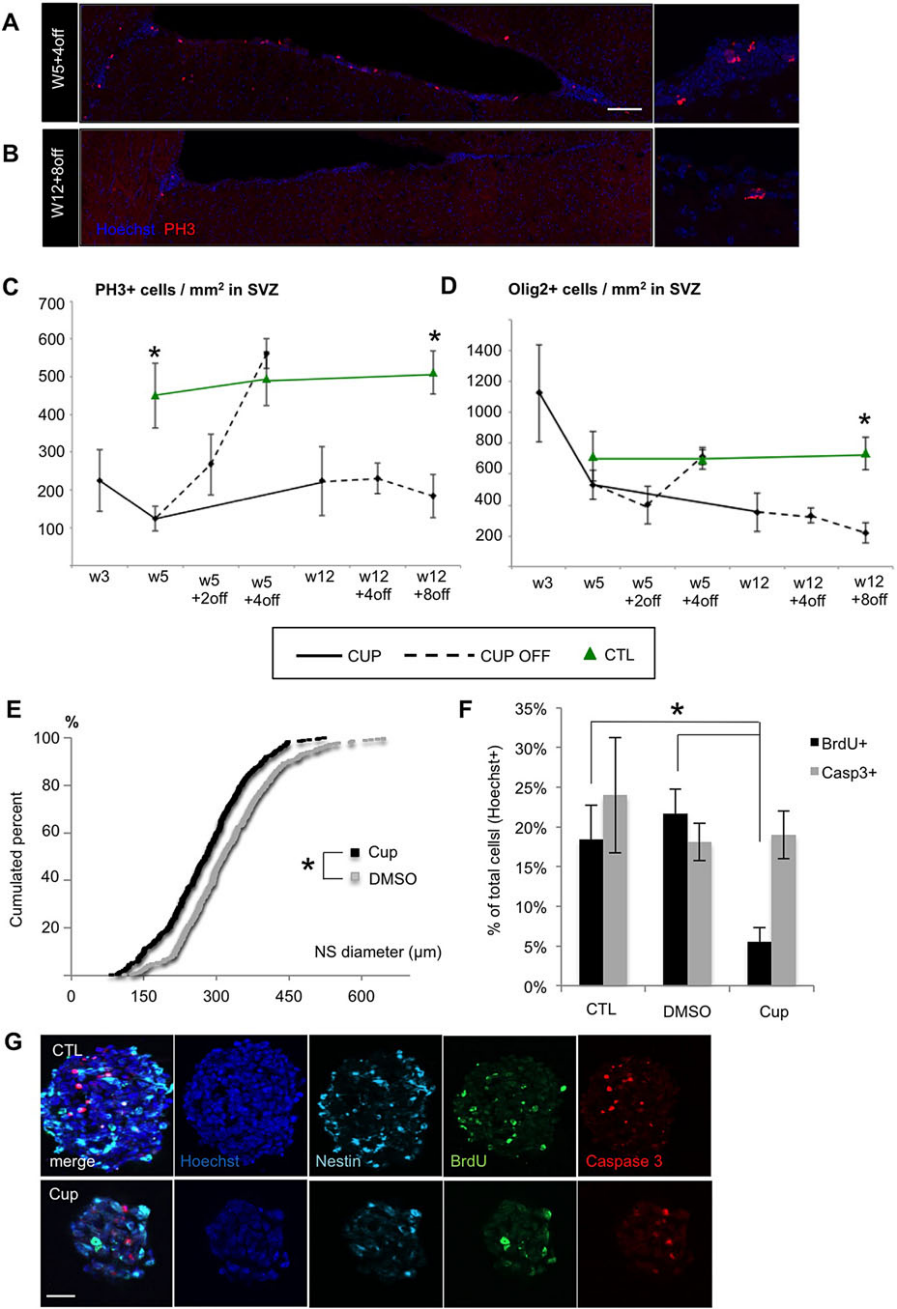
**Effect of long term cuprizone treatment on cell proliferation and oligodendrocyte production in the SVZ.** (A,B) PH3 immunolabeling in SVZ in the (A) acute and (B) chronic model at the end point of recovery (W5+4off and W12+8off respectively). Note the drastic reduction of cell proliferation in the lateral wall of the ventricle in the chronic model, even after 8 weeks recovery (B). (C,D) Quantitative analysis of (C) PH3+ and (D) Olig2+ cells in the SVZ of cuprizone-treated (CUP) and control (CTL) mice. Dotted lines symbolize periods without cuprizone (after cuprizone removal; CUP OFF). (E-G) Effect of cuprizone in the neurosphere forming assay. Neurospheres are slightly smaller (E) in presence of 20 µM of cuprizone (Cup) in the culture medium compared to control (DMSO). (G) BrdU and Caspase 3 immunolabeling on neurosphere and quantitative analysis (F) showing that BrdU incorporation is reduced in presence of cuprizone. Note in G the size reduction of neurosphere in presence of cuprizone. Scale bars in A and B, 100 µm; in G, 25 µm. Error bars=s.e.m.; *P<0.05.

Attempting to understand the reasons why SVZdNP stop being recruited to CC in the chronic model of demyelination, we next examined V-SVZ cell proliferation in acute and chronic models. We found that cell proliferation in V-SVZ was affected by cuprizone treatment, with a significant 2 folds decrease in the number of PH3+ cells compared to untreated controls, as long as cuprizone was administered (Fig. 8A-C). In the acute model, cell proliferation quickly recovered control levels as soon was cuprizone was removed (Fig. 8C). By contrast, in the chronic model, cell proliferation remained low even 8 weeks after mice were returned to normal chow (Fig. 8A-C).

Interestingly, the presence of increased numbers of OPC (Olig2+ cells) in the SVZ was first detected during the initial phase of demyelination (W3), but their density dropped below control levels at W5 to finally return to control levels at W5+4off (Fig. 8D). These data suggest a transient burst of OPC production in SVZ. Again, when cuprizone was maintained for 12 weeks, OPC density in the SVZ remained very low compared to control levels even 8 weeks after drug removal (219±65 versus 734±105 Olig2+ cells/mm^2^ in cuprizone-treated versus control mice; *P*=0.017; Fig. 8D), consistent with the low efficiency of YFP cell recruitment reported above in the context of chronic cuprizone insult (Fig. 7B).

**Fig. 9.**
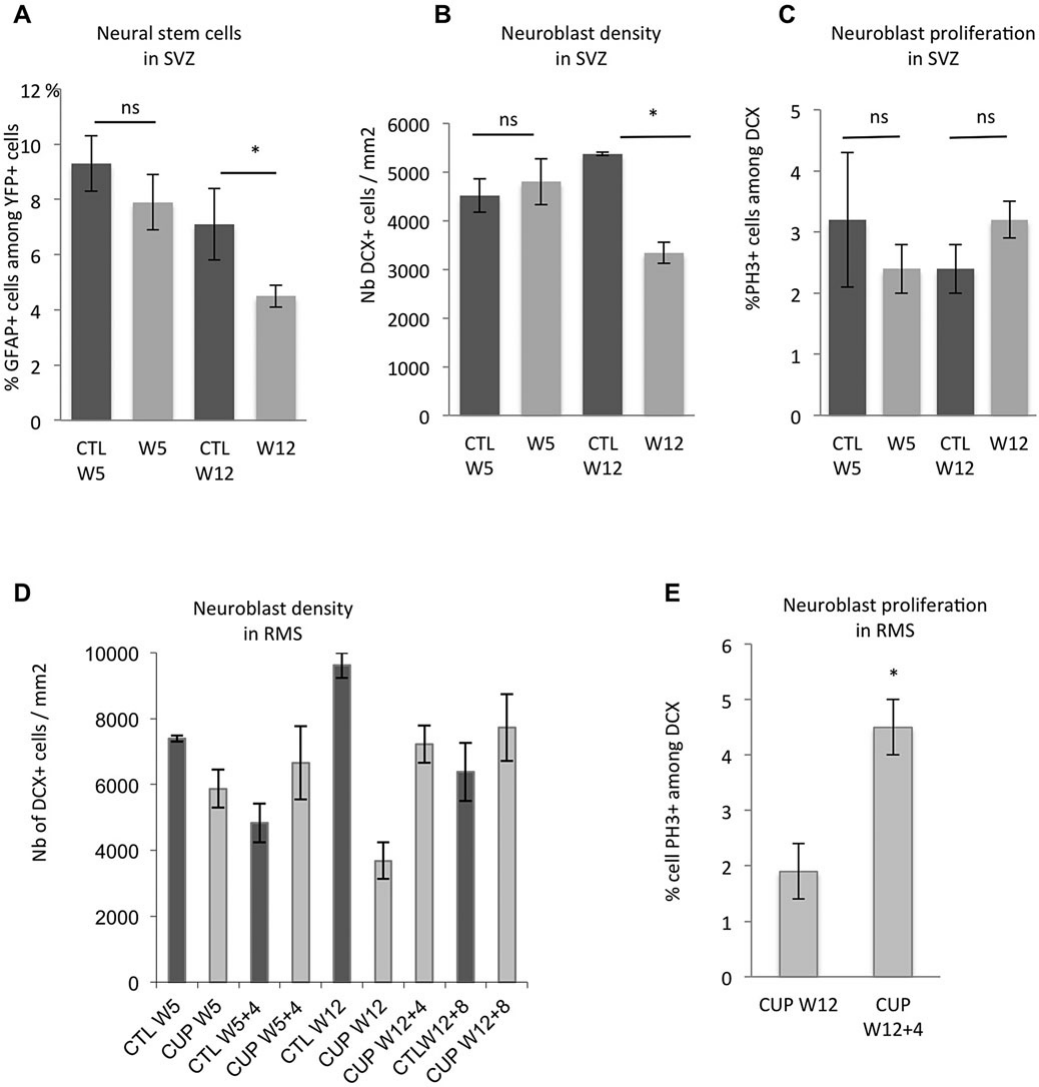
**Effect of long-term cuprizone treatment on NSC and neuroblasts.** (A) YFP+GFAP+ co-labeling in SVZ shows a decrease in the proportion of NSC among YFP+ cells after 12 weeks but not 5 weeks cuprizone feeding. (B) Neuroblast density in SVZ assessed by doublecortin (DCX) immunolabeling. (C) Neuroblast proliferation in the SVZ showed by the proportion of DCX+ cells in M phase (PH3 labeling). Cuprizone does not affect neuroblast proliferation. (D) Neuroblast density in RMS at the entry of olfactory bulb. Olfactory neurogenesis is transiently affected after 12 weeks cuprizone treatment. (E) Proliferation level in neuroblasts in distal RMS after long-term cuprizone treatment. Note the two-fold increase in the proportion of dividing neuroblasts between W12 and W12+4off. *P<0.05; ns, non significant.

We then examined the effect of long-term cuprizone treatment on NSC. In NestinCre^ERT2^-YFP mice, the proportion of GFAP+ cells among YFP+ cells in the V-SVZ was significantly decreased by 37% at W12 compared to control group (4.5±0.4% versus 7.1±1.3%; *P*=0.04) whereas there was no change after only 5 weeks cuprizone feeding (Fig. 9A). These results suggest that NSC are affected by long-term cuprizone administration. Neuroblast density in SVZ was also decreased by 30% (Fig. 9B) at W12. As a consequence, the number of neuroblasts reaching the olfactory bulb at W12 was also drastically impaired (Fig. 9D). However, neuroblast proliferation in SVZ was not significantly different between control and cuprizone-treated mice nor between short- and long-term cuprizone treatment (Fig. 9C). Thus, it seems that neuroblasts themselves are not affected by cuprizone and the lower neuroblast density observed at W12 is a consequence of NSC deficiency and decrease in progenitor cell proliferation. Beside, the drop of olfactory bulb neurogenesis at W12 was only transient because of a compensatory burst in neuroblast proliferation within the distal RMS between W12 and W12+4off (Fig. 9D,E).

Altogether, these results suggest that long-term cuprizone treatment depletes NSCs and reduces progenitor cell proliferation thus leading to SVZ aplasia.

Such SVZ aplasia could either be the result of an exhaustion of the pool of SVZ progenitors due to chronic demyelination, and/or may reflect a direct toxic effect of the compound on SVZ progenitors, both possibilities being non-mutually exclusive. In order to address this question, we cultured SVZ progenitors using the neurosphere assay and examined the effect of cuprizone on cell proliferation and cell death. Addition of cuprizone to the culture medium after the first neurosphere passage led to a slight but very significant reduction in neurosphere diameter (Fig. 8E; *P*<0.001) consistent with reduced cell proliferation. BrdU addition in the culture medium confirmed that cell proliferation was indeed markedly decreased in presence of cuprizone (Fig. 8F,G; *P*<0.001). By contrast, cell survival was not affected as revealed by the absence of increase in the proportion of Casp3+ cells in neurospheres (Fig. 8F,G). These experiments suggest that cuprizone may directly affect SVZ progenitors by inhibiting their proliferation but not survival.

By contrast, the presence of cuprizone in the culture medium did not change the proportion of neurons, astrocytes and oligodendrocytes generated after growth factors withdrawal. It did not affect either maturation of cells within the oligodendrocytic lineage. Thus, cuprizone has no direct impact on cell fate nor on oligodendrocyte maturation (supplementary material Fig. S2).

Altogether, our *in vivo* and *in vitro* results led us to propose that reduced number of NSC, reduced transit amplifying progenitor cell proliferation and reduced OPC generation in SVZ after long-term cuprizone treatment may prevent SVZdNP from contributing efficiently to the repair process.

## DISCUSSION

Several recent studies demonstrated that beside pOPC, progenitors located in the V-SVZ could also be mobilized after demyelination and generate new oligodendrocytes (Jablonska et al., 2010; Menn et al., 2006). Although their contribution to myelin repair is assumed to be minor even negligible compared to pOPC, the relative participation of these two sources of cells displaying markedly different properties to myelin regeneration in different pathological contexts has never been truly assessed. NSC in V-SVZ are endowed with long-term self-renewal potential and reside in a spatially restricted niche. By contrast, pOPC are widespread in the whole brain and have a restricted self-renewal potential. Therefore, we hypothesized that SVZdNP would play a minor role in myelin repair when demyelination is acute and localized in regions remote from SVZ, but might represent a very useful source in the context of chronic demyelination when the pool of pOPC can be exhausted, particularly in areas close to SVZ.

Strikingly and contrary to our predictions, after acute demyelination SVZdNP play a major role in the regenerative process (at least equivalent to pOPC), while in the chronic model they contribute minimally compared to pOPC. Our results unexpectedly revealed that long-term cuprizone-induced demyelination triggers an aplasia of the SVZ that ultimately prevents any sustained contribution of SVZdNP to myelin repair in this chronic model.

By contrast, in agreement with our initial hypothesis our work points out a regionalization in the respective mobilization of SVZdNP and pOPC. After acute cuprizone-induced demyelination we observed a prominent recruitment of SVZdNP in anterior CC while the degree of pOPC mobilization was more pronounced at more posterior levels. The rostral level of analysis (see Fig. 1) corresponds to the most productive area of the SVZ, by contrast to the caudal area where SVZ is less active (Doetsch et al., 1999). Interestingly, such regionalized recruitment and strong remyelination potential of SVZdNP after acute demyelination have been pointed out by a very recent study (Xing et al., 2014). Since SVZdNP proliferation does not differ in anterior and posterior areas, higher cell density in anterior CC is most probably due to more efficient recruitment from the adjacent niche. Consistent with this hypothesis, Xing et al. (2014) also described highest SVZdNP density in the CC adjacent to the dorso-lateral corner of the SVZ after acute demyelination. Intriguingly, although SVZdNP are known to be able to migrate extensively along white matter fiber tracts (Cayre et al., 2006), in this demyelination context they do not seem to travel long distances along the antero-posterior axis after entering the CC and thus they remain regionalized. This may be explained by their very rapid differentiation into oligodendrocytes as shown here. Contrary to Xing et al. (2014) who described early recruitment of SVZdNP but no obvious oligodendrogenesis before 4 weeks of cuprizone treatment, we observe a rather low recruitment of SVZdNP at W3 that amplifies considerably between W3 and W5, concomitantly with quick adoption of oligodendrocytic fate (1/3 and 2/3 of YFP+ cells at W3 and W5 respectively) and maturation into CC1+ oligodendrocytes (1/3 and 1/2 of YFP+Olig2+ cells at W3 and W5 respectively). Altogether these results suggest that production of mature oligodendrocytes by SVZdNP becomes subsequent between W4 and W5.

The regionalized contribution of SVZdNP with very strong mobilization in the anterior part of the CC is remarkable in the light of previous studies describing differential susceptibility of the CC to cuprizone-induced demyelination. Indeed, the caudal part of the CC is more affected than the anterior part by cuprizone treatment (Steelman et al., 2012; Stidworthy et al., 2003; Wu et al., 2008; Xie et al., 2010). Remarkably, areas with limited extent of demyelination correlate with high SVZdNP density. The precocious and substantial recruitment of SVZdNP in the anterior CC followed by their rapid progression towards maturation into myelinating oligodendrocytes may contribute to limit demyelination extent and/or accelerate myelin repair. Besides, the fraction of SVZdNP that remain undifferentiated may also exert a protective influence and contribute to limit demyelination. Indeed, recent studies outlined a bystander immunomodulatory effect of transplanted NSC beneficial for regeneration (Cusimano et al., 2012; Pluchino et al., 2003). Further studies will be needed to address this question.

Since pOPC are evenly distributed in the entire CC, the reasons for the regionalized cell density we observed in the acute model are not clear. Several hypotheses implying either environmental regulation or intrinsic properties can be formulated. The complementary distribution of SVZdNP and pOPC in the CC, also outlined by Xing et al. (2014), may suggest an interplay between these two cell populations in this pathological context. We observed no difference in the proportion of proliferating cells among pOPC between anterior and posterior CC that could explain the regionalization. By contrast, differentiation rate of pOPC is different since the proportion of CC1+ oligodendrocytes among YFP+ cells is doubled in anterior compared to posterior CC at the end of cuprizone exposure (W5). Precocious maturation may thus lead to reduce the pool of immature reactive pOPC. Finally, recent studies suggested that pOPC are not a homogeneous population of cells: notably they can be separated into dividing and non dividing subpopulations of oligodendrocytes in the adult CC (Rivers et al., 2008); pOPC can also differ in their ion channels expression profiles, their excitability properties (Káradóttir et al., 2008; Yuan et al., 2002) and their differentiation potential (Viganò et al., 2013), all these differences possibly defining functional subsets. Thus, a possible uneven composition of pOPC in anterior and posterior CC may also account for the differences observed.

Chronic demyelination is observed in mice exposed to long-term cuprizone administration (Skripuletz et al., 2011). In this model (12 weeks of cuprizone exposure), a progressive depletion of pOPC is described, starting from 6 weeks of treatment (Mason et al., 2004). Our results showed that in spite of their relative reduction in number, pOPC that survive after 12 weeks of cuprizone treatment remain reactive, they proliferate at similar level as after only 5 weeks treatment, and contribute to replenish the CC. Beside, their maturation into CC1+ oligodendrocytes was notably increased in the chronic compared to the acute model (Fig. 3D). This increase in pOPC differentiation could in part compensate for reduced pOPC pool to achieve myelin repair. However, the repair process was incomplete even after 8 weeks recovery, Olig2 cell density remaining below control values and remyelination being only partial (as illustrated in supplementary material Fig. S1).

Our initial hypothesis predicted that in a chronic context SVZdNP would compensate for pOPC depletion and predominantly contribute to myelin repair. However, by contrast to the massive recruitment of SVZdNP observed during the initial phase of demyelination, when cuprizone was maintained for 12 weeks the number of V-SVZ-derived cells in the CC drastically decreased. These results suggest that oligodendrocytes generated from early-recruited SVZdNP do not survive under sustained cuprizone exposure. Even more surprising, SVZdNP were no longer recruited to the demyelinated CC even after cuprizone removal, as if the V-SVZ did not respond anymore to injury signals. We indeed evidenced an irreversible loss of cell proliferation in V-SVZ caused by long-term cuprizone exposure in the chronic cuprizone model. Neural stem/progenitor cells in V-SVZ and pOPCs display very different physiological and metabolic properties that may underlie their different susceptibility to cuprizone. In particular, normal mitochondrial function has been reported to be crucial to SVZ cell proliferation (Lee et al., 2013; Ramos-Mandujano et al., 2014) and the main presumptive mechanism of action of cuprizone is mitochondrial dysfunction.

Stem cell niches are sensitive to pathological processes. Likewise, SVZ cell proliferation is most often activated after acute insults such as trauma, ischemia or focal demyelination induced by lysolecithin injection (for review see Miles and Kernie, 2006). By contrast, in neurodegenerative pathologies, data are more ambivalent with sometimes conflicting results describing either up or down regulation of SVZ cell proliferation (for review see Curtis et al., 2012). Interestingly, in experimental autoimmune encephalomyelitis (EAE), a mouse model of MS with an acute phase followed by remission and relapses, SVZ exhibits a transient increase in proliferation during acute EAE that is lost during the chronic phase despite persistent injury (Rasmussen et al., 2011). Likewise, in the chronic cuprizone model we observed SVZ cell proliferation exhaustion and NSC depletion that may be in part attributed to sustained demyelination. To note, a recent study revealed that actively dividing neural stem cells present in the adult subependymal zone have a limited self-renewal potential and get exhausted after generating several waves of strongly expanding progeny (Calzolari et al., 2015). This may contribute to SVZ exhaustion we observed in the chronic model. However, we also showed a direct inhibitory effect of cuprizone on SVZ cell proliferation in culture. Thus we cannot exclude that the failure of SVZ to contribute to the repair process in the chronic model is not due specifically to the effect of cuprizone on SVZ cell proliferation rather than to chronic demyelination that would exhaust NSC pool. It would be interesting to test SVZ potential in another chronic demyelinating model.

Altogether our work sheds new light on the role of adult NSC in myelin repair and shows that pOPC should not be considered as the only possible source of cells for myelin regeneration. It thus opens new therapeutic perspectives for diseases such as MS. In the adult human brain SVZ is the largest germinal region that continues to generate cells along life (Wang et al., 2011). Interestingly, post-mortem analysis of MS patients' brain showed an increase in cell proliferation and in the number of progenitors in SVZ even though the average of disease duration since onset was 28 years, suggesting that prolonged exposure to repetitive inflammatory insults did not fully exhaust SVZ proliferation potential (Nait-Oumesmar et al., 2007). These observations raise hope for the development of alternative therapeutic strategies.

## MATERIALS AND METHODS

All experimental and surgical protocols were approved by the ethic committee for animal experimentation (reference 31-051022012).

### Animals and treatments

PDGFRa-creER^T2^ (Rivers et al., 2008) and Nestin-creER^T2^ (Lagace et al., 2007) transgenic mice were used to trace pOPC and SVZdNP respectively. OPC present in SVZ are not labeled in PDGFRa-creER^T2^ mice (Rivers et al., 2008). Homozygous or heterozygous Cre mice were crossed with homozygous R26R-YFP (Srinivas et al., 2001) or mTmG (Muzumdar et al., 2007) reporters to generate double-heterozygous offspring for analysis. These mice will be referred to as PDGFRaCre^ERT2^-YFP, NestinCre^ERT2^-YFP and NestinCre^ERT2^-mTmG.

Six week old mice were injected for 5 consecutive days with tamoxifen (180 mg/kg) to induce recombination and cell labeling. Cuprizone treatment (0.2% in food) started 2 weeks after the end of tamoxifen injections and lasted for either 5 weeks (acute model) or 12 weeks (chronic model). Mice were sacrificed after 3 (W3), 5 (W5), 12 (W12) weeks cuprizone treatment or 2 (W5+2off), 4 (W5+4off and W12+4 off) or 8 (W12+8off) weeks after cuprizone removal (Fig. 1A,B).

### Tissue preparation and immunohistochemistry

Serial coronal cryosections 20 µm thick of brains fixed with paraformaldehyde 4% were performed. The primary antibodies used are listed in supplementary material Table S1.

### Microscopy and quantification

Images were captured with a Zeiss apotome system (20× objective) and a 780 confocal (40× objective). Confocal z stacks with 1 µm and 0.5 µm increments were performed respectively. CC was analyzed in two antero-posterior locations: above the anterior SVZ (Bregma +0.5 to +1) and posterior to the fornix (Bregma −0.3 to −0.8) (Fig. 1C). Sections from at least three mice were analyzed at each time point. For each mouse, four sections in each location were analyzed. The area of interest was delineated and the cells of interest counted using either Axiovision or Zen softwares (Zeiss).

### Primary cultures of neurospheres and proliferation and differentiation assay

Neurospheres were produced as previously described (Durbec and Rougon, 2001). Cuprizone 20 µM (or DMSO 0.1% for solvent alone) was added to the culture medium after the first passage. Neurospheres were then passaged once a week; before each passage, neurosphere number and diameter were measured. Proliferation and apoptosis were examined after BrdU (10 µM added for 4 h in the culture) and activated Caspase3 labeling respectively. The percent of BrdU+ and Caspase3+ cells over total number of cells (nuclei labeled by Hoechst33342, 1/1000) was calculated. Neurosphere cell fate and maturation analyses were performed 2, 4 and 6 days after growth factor removal using specific markers (supplementary material Table S1).

## Supplementary Material

Supplementary Material
