# Region and dynamic specificities of adult neural stem cells and oligodendrocyte precursors in myelin regeneration in the mouse brain

**DOI:** 10.1242/bio.016980

**Published:** 2016-02-15

**Authors:** Béatrice Brousse, Karine Magalon, Pascale Durbec, Myriam Cayre

There was an error published in *Biol. Open* **4**, 980-992.

The statements regarding funding were incomplete and the following text has now been included in the Funding section:

This work was supported by the Fondation pour la Recherche Médicale, grant number [DEQ20140329501] to P.D.

Biology Open and the authors apologise to the readers for any confusion that this omission might have caused.

