# Supplementary material for: Region and dynamic specificities of adult neural stem cells and oligodendrocyte precursors in myelin regeneration in the mouse brain
Source: Biol Open. 2015 Jul 3;4(8):980–92. doi: 10.1242/bio.012773 (PMC4542288; doi:10.1242/bio.012773)
Supplement: Supplementary Material [file supp_4_8_980__index.html]

Region and dynamic specificities of adult neural stem cells and oligodendrocyte precursors in myelin regeneration in the mouse brain — Region and dynamic specificities of adult neural stem cells and oligodendrocyte precursors in myelin regeneration in the mouse brain — Region and dynamic specificities of adult neural stem cells and oligodendrocyte precursors in myelin regeneration in the mouse brain — Supplementary Material 

# Region and dynamic specificities of adult neural stem cells and oligodendrocyte precursors in myelin regeneration in the mouse brain

## BIO012773 Supplementary Material

- Supplementary Material
