# Supplementary material for: Region and dynamic specificities of adult neural stem cells and oligodendrocyte precursors in myelin regeneration in the mouse brain
Source: Biol Open. 2015 Jul 3;4(8):980–92. doi: 10.1242/bio.012773 (PMC4542288; doi:10.1242/bio.012773)
Supplement: Supplementary Material [file supp_bio.012773_BIO012773supp.pdf]

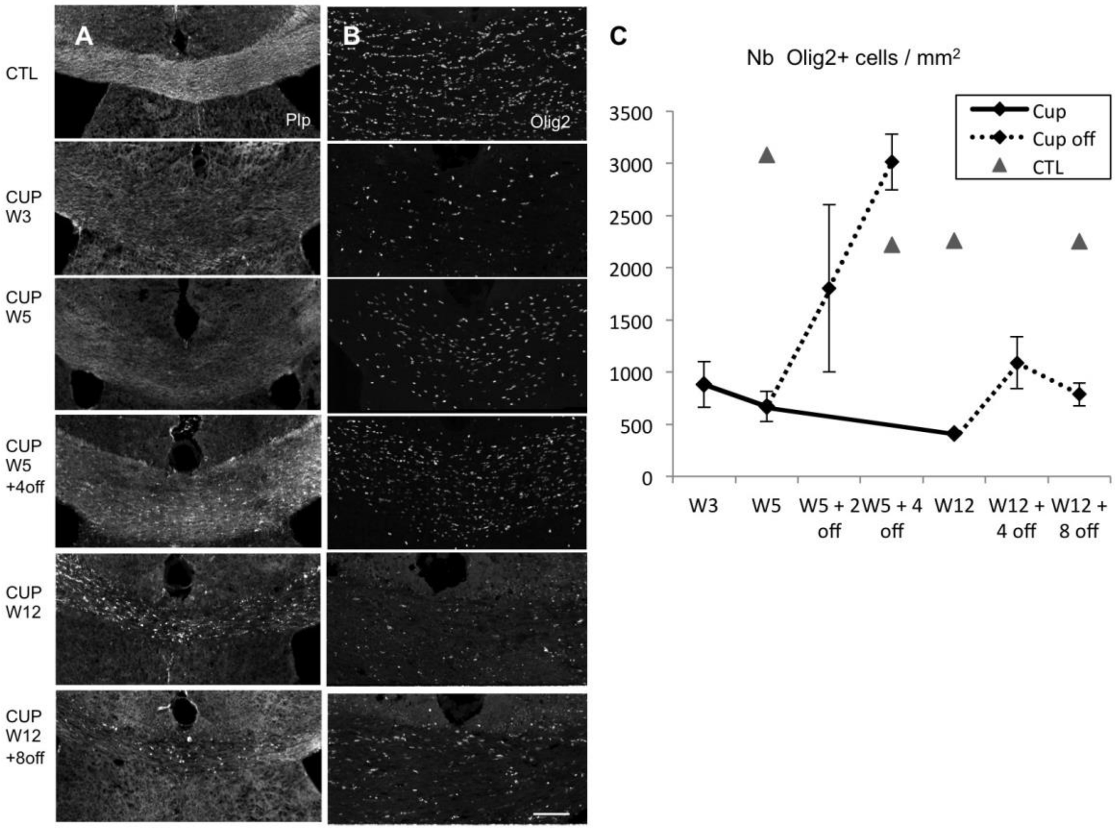

Supplementary Figure 1: Validation of the acute and chronic cuprizone demyelination models in NestinCre^{ERT2}-YFP and PDGFRαCre ^{ERT2}-YFP mice.

A: Cuprizone treated mice exhibited extensive demyelination in CC as revealed by proteolipid protein (Plp) immunolabelling (illustrated here in a PDGFRαCre^{ERT2}-YFP mouse). The homogeneous staining observed in the CC of non-treated mice became patchy and faint after 3 weeks of cuprizone feeding, and almost completely disappeared after 5 weeks. Four weeks after cuprizone removal, Plp staining recovered levels comparable to those of control animals. When cuprizone was maintained for 12 weeks, myelin was absent along axons in the CC but Plp expression was detectable in cell soma. Although myelin staining increased after cuprizone removal it never reached back control levels. B: Variations of Olig2+ cell density in the CC over time (illustrated here in a NestinCre^{ERT2}-YFP mouse). Olig2 is a transcription factor expressed in all the cells of the oligodendrocytic lineage, from progenitors to myelinating cells. C: Quantitative analysis of Olig2+ cell density in the CC of NestinCre^{ERT2}-YFP mice during acute and chronic demyelination. As soon as 3 weeks after starting cuprizone feeding, the number of Olig2+ cells per mm^2 dropped to approximatively one third of control values, remained low as long as cuprizone was maintained, but quickly increased up to control values when animals returned to normal food. By contrast, in the chronic model (i.e. when cuprizone was administered during 12 weeks), Olig2+ cell density in the CC remained below control values even 8 weeks after removal of cuprizone in the food. The effect of cuprizone treatment described above as well as its dynamic was similar in NestinCre^{ERT2}-YFP and in PDGFRαCre ^{ERT2}-YFP mice (not shown). Scale bars in A and B, 100µm.

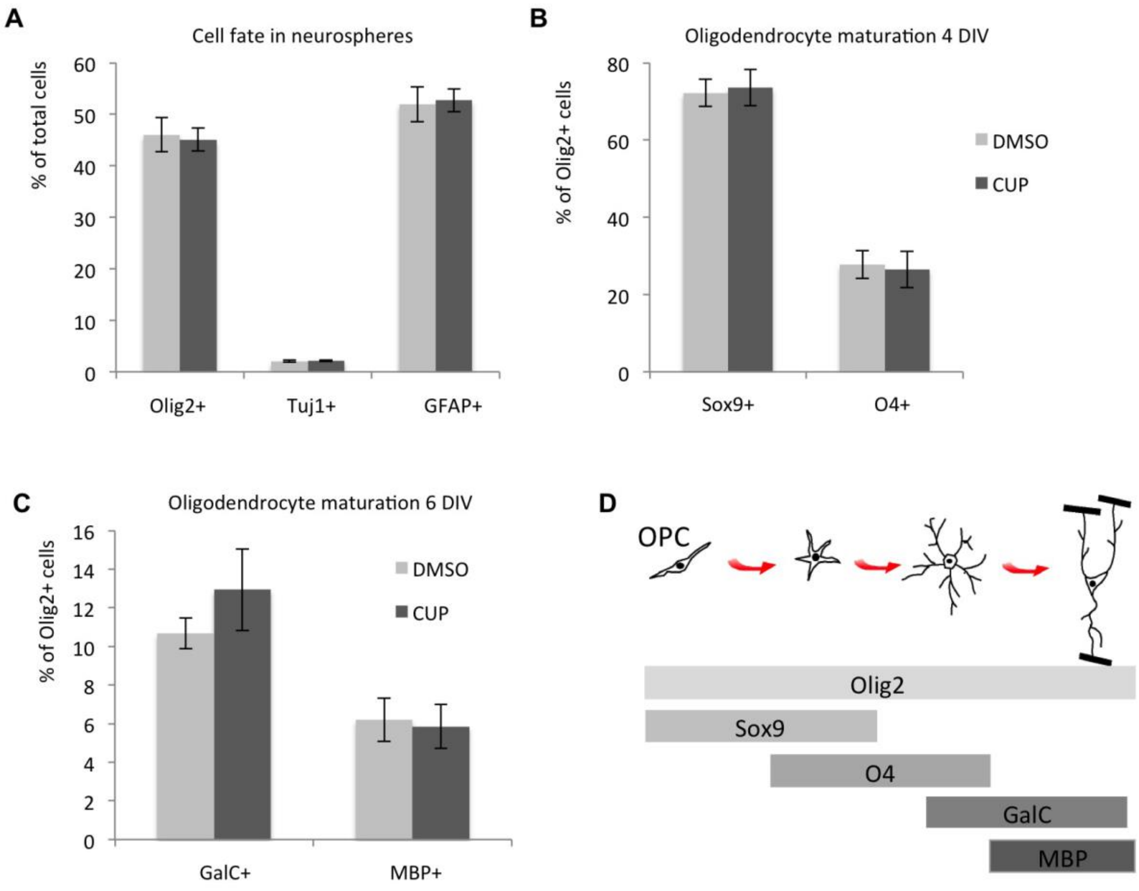

Supplementary Figure 2: Effect of cuprizone on cell fate and maturation in vitro.
A: Neurospheres were grown in differentiation medium for two days, and progenitor differentiation into neurons, oligodendrocytes and astrocytes was analyzed. The presence of cuprizone in the culture medium did not affect cell fate. B,C: Oligodendrocyte maturation was tested after 4 days (B) and 6 days (C) in the differentiation medium. The presence of cuprizone in the culture medium did not influence maturation. D: Diagram showing the expression of the different markers used to monitor maturation process.

Table S1: Antibodies used in this study

Antibody	host species	Concentration	Supplier
In vivo experiments			
GFP	Chiken	1/1000	Aves Labs
Olig2	Rabbit	1/500	Chemicon
PDGFRa	Rat	1/250	Chemicon
NG2	Rabbit	1/200	Chemicon
CC1	Mouse	1/500	Calbiochem
MBP	Mouse	1/500	Chemicon
Plp	Mouse	1/500	Millipore
GFAP	Mouse	1/1000	Sigma
DCX	Goat	1/250	Santa Cruz
Caspr/paranodin	Rabbit	1/500	L51, gift from Dr Goutebroze
PH3	Rabbit	1/250	Upstate
Ki-67	Mouse	1/200	BD Pharmingen
In vitro experiments			
Cleaved caspase 3	Rabbit	1/200	Cell Signaling
BrdU	Rat	1/300	AbCys
Olig2	Rabbit	1/1000	Chemicon
Tuj1	Mouse	1/1000	Eurogentec
O4	Mouse	Supernatant 1/4	Hybridoma, home made
Gal C	Mouse	Supernatant 1/4	Hybridoma, home made
Sox9	Goat	1/200	R&D
MBP	Rat	1/250	Serotec
